# Dynamics of hepatitis C epidemic among people living with HIV in Estonia based on Estonian HIV cohort study

**DOI:** 10.1186/s12879-021-06521-w

**Published:** 2021-08-10

**Authors:** Kerstin Kase, Radko Avi, Karolin Toompere, Heli Rajasaar, Merit Pauskar, Pilleriin Soodla, Ene-Ly Jõgeda, Kai Zilmer, Irja Lutsar, Kristi Huik

**Affiliations:** 1 Department of Microbiology, Institute of Biological and Translational Medicine, Faculty of Medicine, University of Tartu, Ravila 19, 50411 Tartu, Estonia; 2 Infectious Diseases Clinic, West-Tallinn Central Hospital, Tallinn, Estonia; 3 Department of Epidemiology and Biostatistics, Institute of Family Medicine and Public Health, Faculty of Medicine, University of Tartu, Tartu, Estonia; 4 HIV Dynamics and Replication Program, National Cancer Institute, National Institutes of Health, Frederick, MD USA

**Keywords:** HIV, Hepatitis C, Co-infection, Intravenous drug use, Eastern Europe

## Abstract

**Background:**

Estonia has a typical Eastern European HIV epidemic where the most frequent co-infection is chronic hepatitis C (HCV). We aimed to describe the changes in HCV prevalence, the distribution of HCV genotypes (GT), and HCV treatment in Estonian people living with HIV over 15 years.

**Methods:**

We used data of subjects included to the Estonian HIV Cohort Study (E-HIV) before 31st of December 2015. We compared two time periods—first, 1st of January 2000 to 31st of December 2008 when the HIV epidemic was mostly spreading among people who inject drugs (PWID) and second, 1st of January 2009 to 31st of December 2015 when HIV started to emerge to the general population.

**Results:**

Of 4422 HIV positives 3708 (84%) had information about their HCV serostatus; 2706 (61%) were HCV seropositive, of latter 1625 (60%) were HCV RNA positive, 239 (9%) had their HCV GT determined, and 141 (5%) received treatment for HCV. The dominating subtypes were 1b (42%) and 3a (37%) followed by 1a (16%), and the few cases of 2 (1.5%). HCV prevalence was 1.5 times (95% CI 1.4–1.6) higher in subjects diagnosed with HIV in first as compared to those diagnosed in second period (84% vs 56%, respectively). There were more men and the median age at HIV diagnosis was lower in HIV/HCV co-infected than in HIV mono-infected patients (70% vs 47% and 24 years vs. 30 years, respectively; both p < 0.001).

**Conclusion:**

There is a decrease in HCV prevalence but it remains high among HIV positive PWID, suggesting that there is need for improvement of harm reduction programs among PWID.

## Introduction

Chronic infection by hepatitis C virus (HCV) is the most common co-infection of HIV positive patients especially in those who report intravenous drug use (IVDU) [1–3]. HIV/HCV co-infection plays an important role in the prognosis of HCV infection considering that co-infected patients develop end-stage liver disease with cirrhosis and hepatocellular carcinoma at a younger age than HCV mono-infected patients [4–6]. HIV/HCV co-infection may increase the risk of neurocognitive impairment [7], and increase the risk of cardiovascular disease [8]. To decrease the progression of liver disease, and to improve the treatment outcome, HCV should be treated in all patients. Nevertheless, among HIV/HCV co-infected patients, HCV treatment rates only from 5 to 40% worldwide. An increase in all treatment initiations has been observed after 2012 due to the arrival of the direct-acting antivirals (DAAs) ([9, 10]). Interferon-free regimens with the DAAs are available in Estonia since January 2016 but only for patients with METAVIR score ≥ F2.

The prevalence of HCV varies by country. The HIV cohorts that mostly consist of men having sex with men (MSM) (e.g. Western Europe, North America) have HCV rates between 5 and 20% while cohorts in which IVDU predominate (e.g., countries of the former Soviet Union (FSU)) have very high rates ranging from 40% up to 90% [11, 12]. This is understandable because HCV is predominantly transmitted parenterally and HCV epidemic usually precedes HIV epidemic. Estonia is not an exception as HCV epidemic among people who inject drugs (PWID) emerged already in 1990s; almost decade before an outbreak of HIV infection [13].

The Estonian concentrated HIV epidemic is typical for countries of FSU [14, 15]. It started in August 2000 predominantly among PWID. Although the HIV prevalence is steadily declining in recent years, Estonia still has one the highest number of newly diagnosed HIV infections in European Union [16].

In countries that have introduced comprehensive preventive strategies for PWID, the prevalence of HIV/HCV co-infection has declined [17]. For example, in France and in Spain the prevalence of HIV/HCV co-infection has decreased during the last decade due to harm reduction policies [18, 19]. In Estonia, the National Institute for Health Development in collaboration with the Ministry of Social Affairs organises harm reduction programs: opioid substitution therapy, syringe exchange programs, education on safer injection practices, overdose prevention, counselling and testing for HIV. The implementation of harm reduction programs in Estonia have been introduced gradually since 1997 starting with needle and syringe exchange programme. In 1999 opioid substitution therapy was introduced, directly observed treatment was initiated in 2010, and since 2013 opioid overdose prevention with naloxone is available [20, 21].

Regardless of all efforts, among Estonian and Ukrainian HIV infected key populations (PWID and their sexual partners, MSM, sex workers, prisoners) currently up to 80% are reported of being HCV seropositive [11].

Considering the absence of a detailed description and dynamic data of HIV/HCV co-infection in an Eastern European HIV epidemic, we aimed to describe the change in HCV prevalence along with the distribution of HCV GTs, and treatment in Estonian people living with HIV (PLWH).

## Methods

### Study design

This was a cross-sectional study. The data was collected using Estonian HIV Cohort Study (E-HIV) which is a web-based database established in 2009 and comprises HIV positive subjects aged > 16 years since 1992[15]. In Estonia, all HIV positive patients are treated by infectious diseases specialists in five HIV treatment centres (West-Tallinn Central Hospital, Tartu University Hospital, Ida-Virumaa Central Hospital, Narva Hospital and Pärnu Hospital) and in all four prisons. Participation in E-HIV is offered to all HIV positive subjects at the first visit to infectious disease specialist. The participation in E-HIV is voluntary and a written informed consent is requested from each participant. Around 1–5% of patients refuse to participate in E-HIV (personal communication by treating physicians). The data are first entered during the first visit and then updated at each visit to the physician. Subjects infected before 2009 were entered retrospectively. E-HIV collects sociodemographic (sex, age, route of HIV transmission), clinical data (cART history, opportunistic diseases, comorbidities including presence of HCV infection) and outcome together with date and reason of death as described elsewhere [15]. Based on Estonian HIV diagnosis and treatment guideline, all HIV-positive patients are routinely tested for HCV since the year of 2000, the beginning of HIV epidemic spread in Estonia among IVDU. HCV genotyping is available in Estonia since 1997.In this study we have compared two time periods—first, from 1st of January 2000 to 31st of December 2008 when the HIV epidemic was mostly spreading among PWID containing information of 2780 HIV-positive subjects and second, from 1st of January 2009 to 31st of December 2015 when HIV started to emerge to general population containing information of 1642 HIV-positive subjects.

### Study population

We made data extraction on the 1st of August 2016 and included all HIV positive patients who have been entered into database during the study period. The following information was analysed: sex, date of birth, date of the first positive HIV test, HCV seropositivity and/or HCV PCR positivity and/or viral load year together with time of the first positive HCV test, self-reported route of HIV transmission, HCV genotype and treatment history, CD4+ T cell count and HIV-1 viral load (VL) at the first visit.

### Definitions

HIV positivity was defined as having a confirmed test performed in Estonian HIV Reference Laboratory at West Tallinn Central Hospital using INNO‐LIA® HIV I/II Score (Innogenetics N.V., Gent, Belgium), Bio‐Rad's NEW LAV BLOT I (Bio‐Rad Laboratories Inc., Hercules, CA, USA) and NEW LAV BLOT II (Bio‐Rad Laboratories Inc., Hercules, CA, USA) tests.

HCV positivity was defined as follows: positive HCV antibody test and/or positive HCV RNA and/or determination of HCV genotype and/or treatment for HCV infection. Subjects who had negative HCV antibody test were considered as HCV negative; subjects who had no information about HCV testing were designated as HCV unknown.

The prevalence of HIV/HCV co-infection and the genotypic distribution were calculated based on the year of HIV diagnosis because all HIV positive subjects are always tested for HCV infection whereas date of HCV infection may not be known.

HCV genotype was determined when the patient had medical insurance and was eligible for HCV treatment. In case of repeated HCV testing all GTs were reported with the date of each test. If the GT was determined as GT 1 at first but repeated and determined as 1a or 1b only the latter was reported.

The HIV transmission route was self-reported. Patients with no information were designated as “undetermined”. If route of transmission was reported heterosexual and PWID simultaneously, the latter was considered as most likely route of transmission.

### Statistical analysis

Exact binomial 95% confidence intervals (CI) were calculated for the prevalence of HIV/HCV coinfection for each year. To estimate the change in prevalence of HIV/HCV co-infection between two time periods modified Poisson regression model was fitted and results reported as prevalence ratio with corresponding 95% CI [22, 23]. Sociodemographic characteristics of HIV/HCV co-infected and HIV mono-infected patients were expressed as median and interquartile range (IQR) or proportions (absolute and relative frequencies) and compared using Wilcoxon rank-sum test for continuous and Chi-square or Fisher exact test for categorical variables. To account for the multiple testing, p-values were adjusted using Holm–Bonferroni method. To further examine the association between HIV/HCV coinfection and patient characteristics—age, sex, transmission route and period of HIV diagnosis, adjusted prevalence ratios with 95% CI were calculated from modified regression models.

Analyses were run on complete case basis using only patients with known HCV status.

Statistical analyses were performed in Stata version 14.2 [24].

### Ethical aspects

The authors had no access to the clinical/personal patient data used in our research, all the data was retrieved from E-HIV. E-HIV is approved by the Research Ethics Committee of the University of Tartu (reference number 295/M-21, 26.08.2019), in full accordance with the Declaration of Helsinki and Good Clinical Practice. All subjects in E-HIV signed their informed consent to participate in the cohort.

## Results

### Characterisation of E-HIV database

**Fig. 1:**
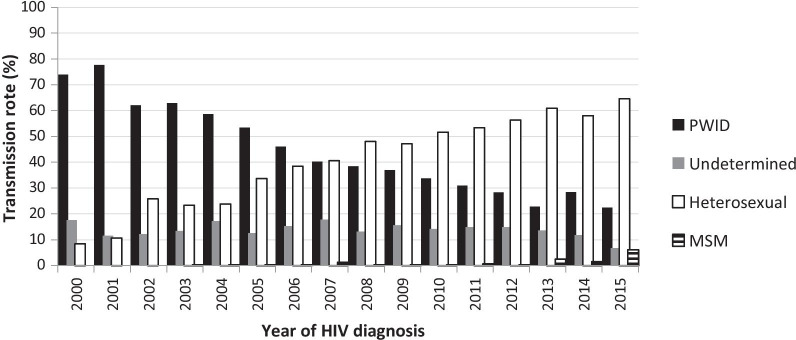
The dynamics of transmission routes in Estonian HIV Cohort Study (E-HIV) from 2000 to 2015. The figure illustrates the patterns of HIV transmission routes from 2000 to 2015 among Estonian PLWH based on the E-HIV data

As of 31st of December 2015 E-HIV included 4422 HIV positive subjects infected during the study period which is 81% of all patients (n = 5439) linked to the care according to the Estonian ARV treatment council (personal communication of Dr. K. Zilmer). The median age at the time of entering care was 25 years (IQR = 21–32). There were more men 64% (2816/4422). The dominating HIV transmission route was IVDU in 48% (2106/4422) followed by heterosexual 38% (1669/4422), 1% (33/4422) in MSM and in 14% (614/4422) the transmission route was undetermined. HIV transmission route changed over the years from being predominantly PWID to predominantly heterosexual transmission (Fig. 1). The median HIV VL at HIV diagnosis was 4.68 log10 copies/ml (IQR = 3.90–5.24) and the median CD4+ T cell count at HIV diagnosis 335 cells/mcl (IQR = 191–510).

### HCV prevalence in Estonian HIV-infected patients

**Fig. 2:**
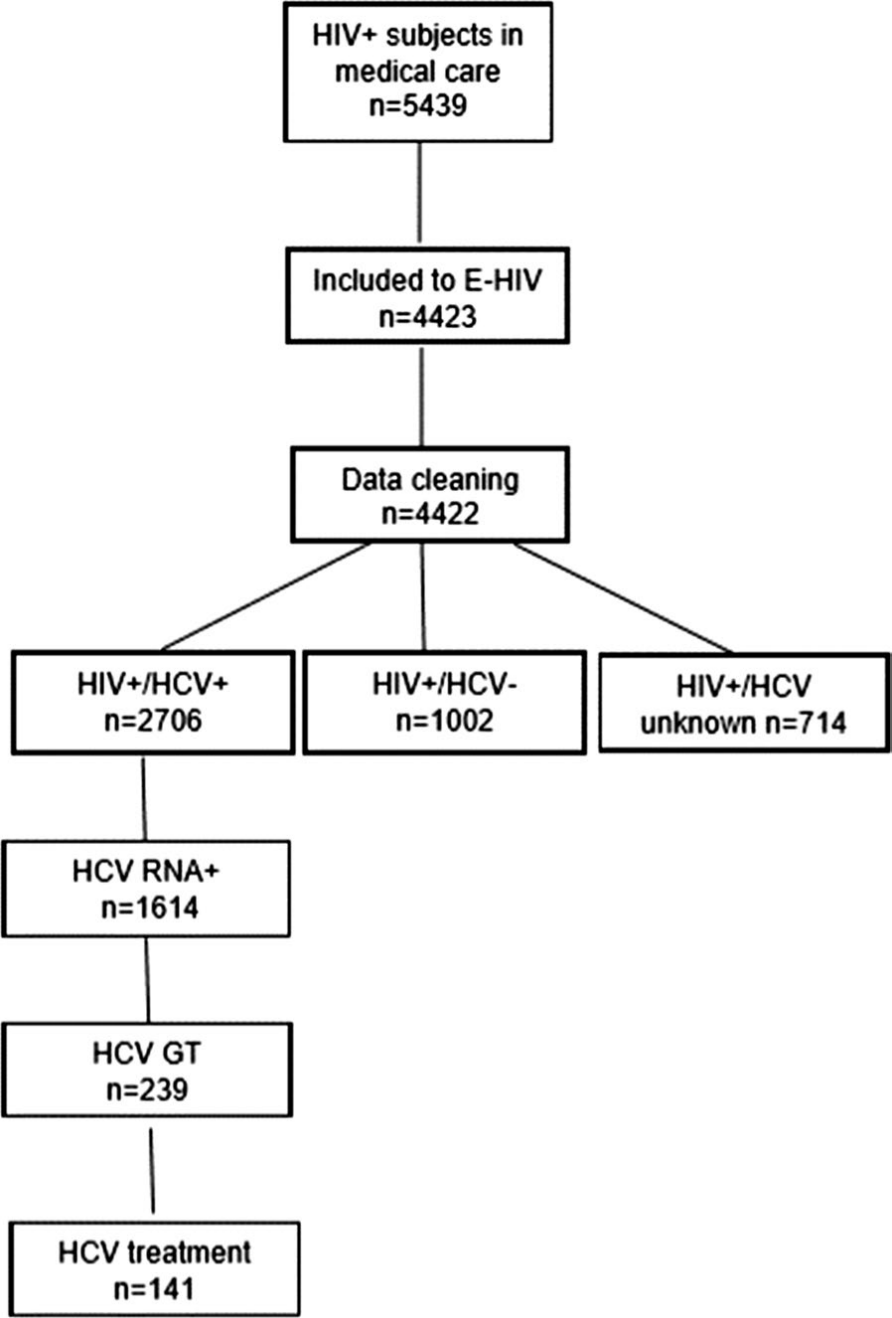
The flow chart of study populations in the Estonian HIV Cohort Study (E-HIV) between 2000 and 2015. The study population included 4422 HIV positive subjects of 5439 patients linked to the care. Of latter, 84% (3708/4422) had information about their HCV serostatus; 61% (2706/4422) were HCV seropositive, 23% (1002/4422) HCV seronegative and 16% (714 /4422) patients had no information about their HCV status. Of HCV seropositive subjects 60% (1625/2706) were HCV RNA positive, 9% (239/2706) had their HCV GT determined and 5% (141/2706) were treated for HCV

Of 4422 subjects, 84% (3708/4422) had been tested for the presence of HCV infection and in total 61% (2706/4422) were HCV positive (Fig. 2). Detailed information on HCV infection is presented in Table 1.

**Table 1:** Analysed groups counted as HCV seropositive

HCV status	Number (%)	2000–2008	2009–2015
HCV ^†^sero+/RNA+	1604 (59.28)	1173	431
HCV sero+/RNA−	204 (7.54)	132	72
HCV sero+/RNA not done	880 (32.52)	614	266
HCV sero+/test done results not known	6 (0.22)	3	3
HCV ^‡^sero−/RNA+	3 (0.11)	3	0
HCV sero unknown/RNA+	7 (0.26)	4	3
HCV sero unknown /RNA not done; received treatment	1 (0.04)	1	0
HCV sero-/RNA not done; received treatment	1 (0.04)	1	0

^†^Sero +: seropositive

^‡^Sero−: seronegative

Of all the HCV positive subjects, 60% (1614/2706) were HCV RNA positive, 9% (239/2706) had their HCV GT determined and 5% (141/2706) were treated for HCV infection (Fig. 2). Among HCV RNA positive individuals, the median HCV VL at first measurement was 6.16 (IQR = 5.60–6.59) log10 copies/ml.

**Fig. 3:**
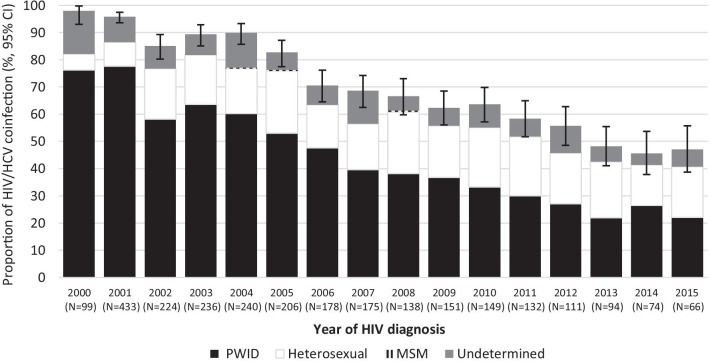
The prevalence of HIV/HCV coinfection in Estonian HIV Cohort Study (E-HIV) from 2000 to 2015 according to HIV diagnosis and the route of transmission. Black bars indicate PWID, white bars heterosexual contact, grey bars undetermined route of transmission and striped bars MSM. Error bars show 95% CI

**Fig. 4:**
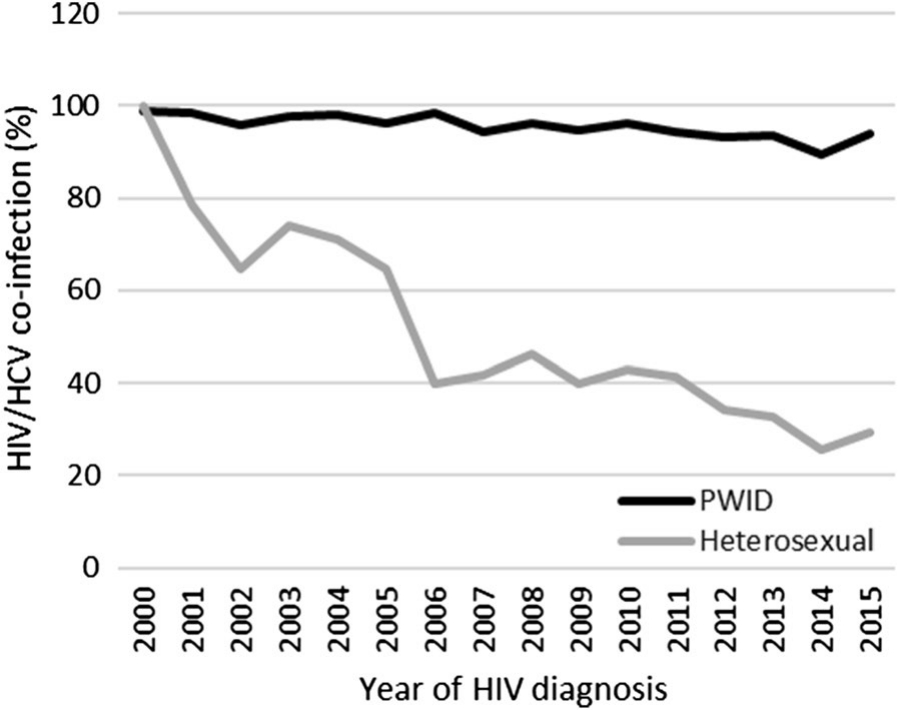
The dynamics of HIV/HCV coinfection in E-HIV from 2000 to 2015 among main transmission groups based on the E-HIV data. Black line indicates PWID and grey line heterosexual contact. Other transmission groups (MSM, unknown) were not analysed

The prevalence of HCV positivity declined over the years from 98% (95% CI 93.0–99.8%) in those diagnosed with HIV infection in 2000 to 47% (95% CI 38.7–55.8%) in those diagnosed in 2015 (Fig. 3). When two time periods were compared, the prevalence was 1.5 times (95% CI 1.4–1.6) higher in subjects diagnosed with HIV during the first compared to those diagnosed in second period (prevalence 84% vs 56%, respectively; p < 0.001). This decline is mainly due to a steady decrease of HCV infection among heterosexually infected subjects while it has remained unchanged (prevalence almost 100%) in PWID during the study period (Fig. 4).

### Comparison of HIV mono- and HIV/HCV co-infected patients

Next, the sociodemographic and clinical characteristics of the study population according to the HCV positivity were compared (Table 2).

**Table 2:** The comparison of the sociodemographic data of the subjects in Estonian HIV Cohort Study (E-HIV) diagnosed with HIV from 2000 to 2015 according to the HCV status

	HIV+/HCV+ (N = 2706)	HIV+/HCV− (N = 1002)	p-value
Age at HIV diagnosis (median, IQR)	24 (20–29)	31 (24–41)	< 0.001
Sex (n,%)			
Male	1907 (70.47)	467 (46.61)	< 0.001
Female	799 (29.53)	535 (53.39)	
HIV transmission route (n,%)			
IVDU	1748 (64.60)	62 (6.19)	< 0.001
MSM	3 (0.11)	23 (2.30)	
Heterosexual	647 (23.91)	773 (77.15)	
Undetermined or missing	308 (11.38)	144 (14.37)	
Year of HIV diagnosis (n,%)			
2000–2008	1929 (71.29)	381 (38.02)	< 0.001
2009–2015	777 (28.71)	621 (61.98)	
HIV VL in Log_10_ at HIV diagnosis (median, IQR) copies/ml	4.68 (3.88–5.21)	4.74 (3.98–5.40)	< 0.001
CD4+ count at HIV diagnosis (median, IQR) cells/mkl	334 (190–510)	321 (190–492)	0.247

Data are given as number (%) of patients; percentages may not always equal 100% because of rounding errors

*IQR* interquartile range, *MSM* men having sex with men, *PWID* people who inject drugs, *VL* viral load

Among HIV/HCV co-infected patients there were more men and the median age at HIV diagnosis was lower than in HIV mono-infected persons. The distribution of transmission route was different in HIV/HCV co-infected patients compared to HIV mono-infected patients—HIV/HCV co-infected subjects were more likely PWID compared to HIV mono-infected who reported being heterosexually infected with HIV (Table 2).

The median age in HIV/HCV co-infected PWID was 25 years (IQR = 21–32) vs 23 years (IQR = 20–28) in HIV mono-infected PWID (p = 0.05). The respective numbers among MSMs were 33 years (IQR = 26–40) vs 27 years (IQR = 23–41), (p = 0.60), for heterosexuals 31 years (IQR = 24–41) vs 26 years (IQR = 21–33), (p < 0.001) and with undetermined transmission route was 32 years (IQR = 25–42) vs 24.5 years (IQR = 20–30), (p < 0.001).

### Factors associated with HIV/HCV co-infection

To investigate whether patient characteristics, age, sex, transmission route and year of diagnosis were associated with HCV positivity, their relative probabilities of belonging to HIV/HCV co-infected or HIV mono-infected group were compared (Table 3).

**Table 3:** Associations between HCV serostatus and patient characteristics in adjusted modified Poisson regression model

HIV + /HCV^+^		
	Adjusted risk ratio^†^	95% CI
Age group		
− 24	1	
25–34	0.95	0.92 … 0.99
35–44	0.79	0.72 … 0.86
45–54	0.54	0.44 … 0.67
55–64	0.36	0.22 … 0.61
65–74	0.23	0.06 … 0.85
Sex		
Female	1	
Male	1.14	1.09 … 1.20
HIV transmission route		
Heterosexual	1	
PWID	1.78	1.68 … 1.89
MSM	0.25	0.09 … 0.71
Undetermined	1.36	1.26 … 1.48
Year of HIV diagnosis		
2009–2015	1	
2000–2008	1.14	1.09 … 1.19

^†^Adjusted to all other variables in the table

**Fig. 5:**
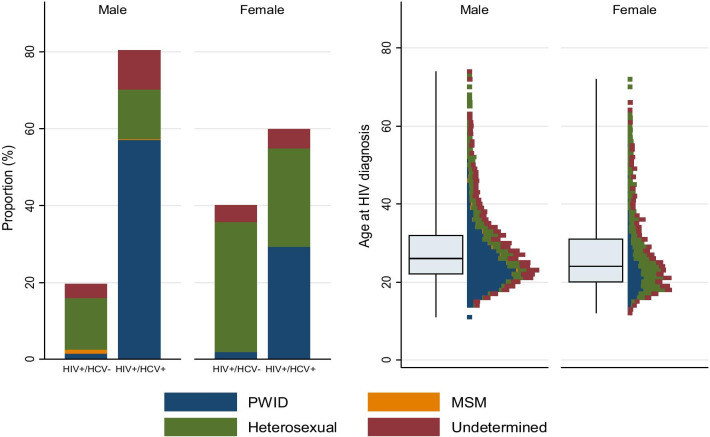
The comparison of the distribution of transmission routes in HIV+/HCV+ and HIV+/HCV− and age at HIV diagnosis between male and female patients in the Estonian HIV Cohort Study from 2000 to 2015. Blue bars indicate PWID, green bars heterosexual contact, red bars undetermined route of transmission and orange bars MSM

The relative risk of being HCV positive compared to HCV negative was higher in men, in subjects diagnosed with HIV in first period, in PWID, and in those with unknown transmission route. Among HIV/HCV co-infected men 71% were reported of acquiring HIV infection via IVDU while among HIV/HCV co-infected women almost equal ratio claimed to be infected via IVDU and heterosexual contact (49% and 43%, respectively). Among HIV mono-infected patients, vast majority of both sexes had acquired HIV infections heterosexually. Undetermined infections were more common among co-infected as compared with HIV mono-infected males whereas there were no differences among females. The age at HIV diagnosis was similar in male and female but men who reported IVDU were younger than men who reported other transmission routes at HIV diagnosis (Fig. 5).

### HCV genotypic distribution

**Fig. 6:**
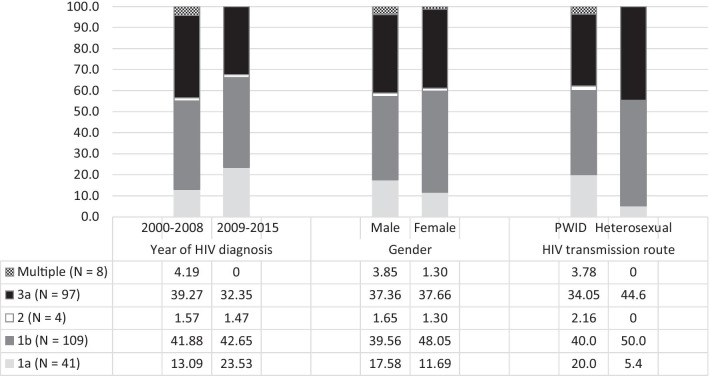
The comparison of the distribution of HCV genotypes between 2000–2008 and 2009–2015 according to period of HIV diagnosis, between heterosexuals and PWID, and between male and female in the Estonian HIV Cohort Study from 2000 to 2015. *Note* Multiple genotypes are 1b + 3a and 1a + 1b

Altogether, HCV GTs were analysed in 239 patients. The dominating subtypes were 1b (42%) and 3a (37%) followed by 1a (16%), and the few cases of 2 (1.5%). In general, HCV genotypic distribution was similar in both periods and among male vs female patients but there were significant differences between heterosexuals and PWID (p = 0.021) (Fig. 6).

### HCV treatment

In total, 5% (141/2706) of co-infected subjects had received HCV treatment. Almost all of them (93%; 131/141) had been treated with pegylated alfa-interferon plus ribavirin (pegIFN + RBV); in 4% of cases telaprevir, boceprevir or simeprevir had been added, and two patients were treated with combination of sofosbuvir and ledipasvir.

## Discussion

To the best of our knowledge, this is the first study looking at the dynamics of HCV infection among PLWH in Estonia, in an area that has experienced sudden increase of HIV infection among PWID in late 1990s early 2000s and that has made several efforts to stop this epidemic. We have made the following observations: (1) the rate of HIV/HCV co-infections among Estonian PLWH is high but the prevalence has decreased over the years most likely due to change in transmission route from intravenous drug use to heterosexual contact; (2) at the same time the prevalence of HCV infection among HIV positive PWID is very high and almost unchanged suggesting low effectiveness of harm reduction programs in preventing HCV infection in that population and continued circulation of HCV in Estonia; (3) the dominating GTs are 1 and 3 similar to other Eastern European countries and there was no significant change in genotypic pattern over time; and (4) only a small number of HIV/HCV co-infected subjects were receiving HCV treatment during the study.

High prevalence of HCV positivity (61%) was not surprising as previous studies in Estonian PWID and other populations have shown rates up to 80–90% [11, 25–27]. In Western European countries HCV positivity among PWID varies depending on the PWID population size of the country and is reaching from 35% in Czech Republic up to 82% in Sweden [27]. Even though both HIV and HCV infection have similar parenteral routes of transmission, HCV is ten times more infectious than HIV and thus it is not surprising that most HIV positive patients are also infected with HCV [28].

We observed that in the early years of Estonian HIV epidemic (2000–2008) the HIV/HCV co-infection rate was higher (84%) than in 2009–2015 when only half of subjects of newly diagnosed HIV positive patients were also HCV positive. This decrease is most likely the result of several factors: (1) the changes in HIV transmission routes from predominantly IVDU to heterosexual contact; (2) implementation of harm reduction programs by the National Institute for Health Development in collaboration with the Ministry of Social Affairs. Harm reduction programmes have been very effective in reducing the prevalence of HIV/HCV co-infection in other countries [18, 19]. They also were effective in reducing HIV infection among PWID in Estonia [15]. It is important to note that despite the reduction of HIV infection among PWID almost all HIV positive PWID are still infected with HCV suggesting that decline of HCV infection among PWID can only been achieved by significant reduction of circulation of HCV in the community. It is hoped that this will happen after new DAAs for treatment of chronic HCV infection are made available to all patients including PWID and HIV/HCV co-infected patients [29, 30]. According to our study as of end of 2015 only 5% of patients with HCV were receiving anti-HCV treatment similar to other Eastern European countries [10, 31].

In this study there is a notable HCV positivity among heterosexually infected PLWH (24%) despite that HCV infection is rarely transmitted by heterosexual contact [32]. These findings are similar to studies from Asia, in which the prevalence of HIV/HCV co-infection varied between 20 and 45% [33]. Overall the biased reporting in our study cannot be excluded. The high prevalence of HCV infection among heterosexual PLWH could be due to the self-reported transmission route, which can reduce the disclosure of some risk groups (e.g. MSM, PWID in women) and will give preference to reporting heterosexual contact as a potential route of transmission. Regrettably self-reporting is almost the only way of collecting data on transmission routes. Furthermore, one should also bear in mind that in E-HIV the patients are asked to report their route of transmission of HIV infection and not of HCV infection. As the HCV epidemic preceded HIV epidemic in Estonia the possible transmission routes for HCV and HIV may differ to some extent and thus some patients who acquired HIV through heterosexual contact might have acquired HCV beforehand via intravenous drug use.

The low percentage of people receiving HCV treatment is of concern. Most likely, reasons for this are as follows: (1) most of the co-infected patients are PWID and do not have Health Insurance; (2) they are active drug users, making them unsuitable for treatment with pegIFN + RBV; (3) long treatment duration, poor tolerability and low sustained virological response to pegIFN + RBV in HIV/HCV co-infected patients (especially in patients with genotype 1). The situation is expected to improve in the future as more DAAs will be reimbursed by the Estonian government. According to Estonian Health Insurance Fund from January 2016 to September 2018 already 299 HIV/HCV co-infected patients have been treated (K Kaal, personal communication, 3st of October 2018) vs 141 patients treated from January 2000 to December 2015.

Some limitations should be noted. First, as mentioned above the self-reporting of the transmission routes may lead to over representation of heterosexual route among co-infected patients. Second, there was a considerable amount (16%) of HIV positive/HCV status unknown individuals. Third, many of our HIV/HCV co-infected patients have information only about their HCV antibody status but no data on HCV RNA or GT that could be due to fact that many of these patients, mostly PWID, have no Health Insurance and thus will not be tested for HCV RNA and GTs. Fourth, E-HIV covers 81% of PLWH in medical care. All the data available from 1992 to 2009 have been recorded into E-HIV retrospectively but it does not capture those who have died or left the country. Despite these limitations, we believe that this study gives an adequate overview of the HIV/HCV co-infection in Estonia.

## Conclusions

In recent years the prevalence of HIV/HCV co-infection has decreased significantly among PLWH in general but still most HIV positive PWIDs are co-infected with HCV. This study suggest that current preventive measures have not been sufficient and that further measures like providing DAAs to all HCV positive subjects should be undertaken to reduce HCV infection among PLWH in Estonia.

## Data Availability

Data file underlying the findings is available from an open access repository for publishing research data from the University of Tartu (DataDOI; https://doi.org/10.23673/re-290).

## Abbreviations

cART: Combination antiretroviral therapy
DAAs: Direct-acting antivirals
E-HIV: Estonian HIV Cohort Study
FSU: Former Soviet Union
GT: Genotype
HCV: Hepatitis C virus
IQR: Interquartile range
IVDU: Intravenous drug use
MSM: Men having sex with men
pegIFN + RBV: Pegylated alfa-interferon plus ribavirin
PLWH: People living with HIV
PWID: People who inject drugs
VL: Viral load
